# Acceptance-based process variables on postpartum well-being and distress: The mediating role of psychological flexibility

**DOI:** 10.1371/journal.pone.0312454

**Published:** 2024-12-20

**Authors:** Alexa Bonacquisti, Emily C. Woodworth, Maria Diaz, Victoria A. Grunberg

**Affiliations:** 1 Department of Clinical Psychology, Philadelphia College of Osteopathic Medicine, Philadelphia, PA, United States of America; 2 Department of Psychology, University at Albany, State University of New York, Albany, NY, United States of America; 3 Department of Psychology and Neuroscience, Temple University, Philadelphia, PA, United States of America; 4 Center for Health Outcomes and Interdisciplinary Research, Mass General Brigham, Boston, MA, United States of America; 5 Harvard Medical School, Boston, MA, United States of America; 6 Division of Newborn Medicine, Mass General Brigham, Boston, MA, United States of America; Alexandria University Faculty of Nursing, EGYPT

## Abstract

**Objective:**

Although up to 20% of women experience postpartum depression and/or anxiety, current interventions are limited. Identifying the processes that impact outcomes can inform and enhance interventions. Our study aims to examine: (1) whether acceptance-based process variables (awareness, acceptance, cognitive defusion, psychological flexibility) were associated with postpartum outcomes; and (2) whether psychological flexibility mediated the relationship between treatment engagement and postpartum outcomes.

**Method:**

Postpartum women (*N* = 266) were recruited online using snowball sampling via social media (i.e., Facebook). They completed a cross-sectional survey of validated and reliable quantitative measures assessing their well-being, emotional distress, and acceptance-based processes.

**Results:**

Hierarchical regressions revealed that women with more awareness (*β* = .13; *p* = .023), acceptance (*β* = .17; *p* = .036), and cognitive defusion (*β* = -.46; *p* < .001) reported better well-being. When adding in psychological flexibility, cognitive defusion (*β* = -.24; *p* = .031) and psychological flexibility (*β* = -.33; *p* = .003) explained variance in well-being. Women with more acceptance (*β* = -.32; *p* < .001) and cognitive defusion (*β* = .52; *p* < .001) endorsed less emotional distress. When adding in psychological flexibility, acceptance (*β* = -.28; *p* < .001), cognitive defusion (*β* = .40; *p* < .001), and psychological flexibility (*β* = .18; *p* = .047) explained variance in emotional distress. Mediation models revealed that psychological flexibility explained the relationship between mental health treatment and well-being (*b* = 3.91, SE = 0.66, CI = [2.69, 5.30]) and emotional distress (*b* = -5.74, SE = 1.08, CI = [-1.26, 3.00]).

**Conclusions:**

Targeting these acceptance-based processes in interventions may help to improve maternal, child, and family outcomes.

## Introduction

Perinatal mood and/or anxiety disorders (PMADs) are common among childbearing women, with an estimated 10–20% of women experiencing perinatal depression and/or anxiety [1–5]. PMADs symptoms can include depressed mood, fatigue, changes in appetite, sleep disturbance, hopelessness, irritability, excessive guilt, suicide ideation and/or thoughts of harming the infant [6, 7]. Anxiety symptoms (e.g., worry, panic, hypervigilance, irritability and intrusive thoughts) are also common [4]. In addition, women may experience a range of other symptoms that contribute to the complexity and severity of these disorders, necessitating careful screening among healthcare providers and connection to appropriate care [8]. When untreated, PMADs are a leading cause of morbidity and mortality among women of reproductive age, resulting in emotional distress and impairments in quality of life and functioning [9, 10]. PMADs can disrupt maternal-infant attachment and bonding, which can, in turn, impact child development [10]. Given this, PMADs must be recognized and addressed as complex, multifaceted disorders that require comprehensive care.

To address the substantial personal and public health concerns associated with PMADs, understanding mechanisms within psychological interventions that can prevent and treat PMADs is needed. Currently, Cognitive Behavioral Therapy (CBT) and Interpersonal Psychotherapy (IPT) are the gold standard treatments for PMADs [11], although most focus on perinatal depression [11–13], and only recently have examined perinatal anxiety [11, 14]. Although these interventions have shown promise, they are limited in several ways. First, these interventions have only exhibited small effects on PMADs symptoms [14] (despite being conducted with small samples–which should inflate effect sizes) [13–15]. These small effects are likely related to the methodological heterogeneity of these trials (e.g., variable delivery, structure, treatment targets) [14, 16, 17]. Second, a significant portion of women do not respond to treatment (~10–40% do not see improvements in depression) [18]. This high rate of non-responders indicates a need to better understand mechanisms of action as well as new ways to address the unique and diverse needs of this population. Finally, current interventions have been primarily tested with homogeneous samples (e.g., White, educated women). In the few studies that have examined CBT or IPT for low-income or minority women with PMADs, results have been mixed [11], suggesting that more tailored and culturally-informed interventions are needed. Given the social impact of PMADs (e.g., stigma, relationship/role changes, access to resources), more accessible interventions that address these challenges and promote values-based living may help enhance efficacy-effectiveness outcomes. Given these limitations, new interventions are clearly needed. An important first step to developing interventions involves understanding whether hypothesized treatment processes and mechanisms are associated with relevant outcomes.

Across third-wave cognitive-behavior therapy approaches (e.g., Acceptance and Commitment Therapy [19] and Mindfulness-Based Cognitive Therapy [20]), psychological flexibility has been shown to be an important mechanism of change. Psychological flexibility is the process of accepting thoughts and feelings and acting consistently with values in the presence of distress [21]. It facilitates adaptation to situational demands, shifts perspectives and behaviors, and increases self-awareness and ability to balance various demands [22]. Psychological flexibility encompasses multiple processes (e.g., present-moment awareness, acceptance, cognitive defusion) that all help to manage external stressors and internal distressing experiences. Because uncertainty, role changes, and conflicting emotions are often germane to the postpartum period, psychological flexibility emerges as an important factor during this time [23]. In fact, psychological flexibility has been shown to protect against postpartum depression and anxiety [24]. Women with PMADs often expect the transition to motherhood to be exclusively positive and joyful, which can lead to feelings of guilt, shame, and/or self-criticism when they have negative thoughts and emotions [25]. These conflicting emotions may be stigmatizing and increase attempts to control and/or avoid internal experiences, thus contributing to psychological rigidity. For these reasons, acceptance-based processes (e.g., awareness, acceptance, cognitive defusion, psychological flexibility) can help women identify and create distance from thoughts and emotions and subsequently engage in valued activities.

Despite the relevance of these processes, only one study, to date, has examined the role of psychological flexibility within postpartum women [24]. They reported that psychological flexibility was associated with reduced depression and anxiety among women at risk for PMADs [24]. In addition, ACT—third-wave behavior therapy that seeks to increase psychological flexibility [26]—has recently been investigated as a treatment for PMADs. In addition to Montiero and colleagues [24], literature has examined the theoretical framework for utilizing ACT with this population [27–29] and other small trials have explored acceptability, feasibility, and preliminary efficacy of ACT for perinatal distress [30–34]. Overall, these studies indicate that ACT may be a promising framework; however, empirical evidence for potential mechanisms of change that can be applied across therapeutic approaches is lacking. A better understanding of which acceptance-based process variables are relevant for both facilitating maternal well-being and reducing emotional distress is needed. Investigating these processes can help inform key mechanisms to target—therefore improving the efficacy and efficiency of current interventions and informing the development of new interventions for perinatal women.

### Study aims

The purpose of the current study was to examine whether acceptance-based process variables help to explain variance in postpartum outcomes. Our aims were two-fold: (1) examine whether awareness, acceptance, cognitive defusion, and psychological flexibility explain variance in well-being and emotional distress among postpartum women; and (2) test whether psychological flexibility mediates the relationship between treatment engagement and postpartum outcomes. Together, findings can inform whether these hypothesized third-wave cognitive-behavioral mechanisms are important to address within interventions for perinatal women.

## Materials and methods

### Design

Using a cross-sectional design, participants provided informed consent and completed four self-report measures online through Qualtrics (Qualtrics, Provo, UT). This survey was anonymous (i.e., no identifying information was collected from participants). Institutional Review Board approval from Holy Family University was obtained prior to initiating study procedures and participants provided electronic written consent. The study was conducted from April 11, 2019 through June 1, 2019.

### Participants

Eligibility criteria were as follows: (1) over age 18, (2) living in the United States, and (3) had given birth within the last year, which is considered the postpartum period [35]. We excluded participants if their delivery did not result in a live birth—loss is a unique emotional experience (e.g., grief) that warrants its own investigation.

### Measures

#### Socio-demographics

Participants reported their demographics including age, income, race, ethnicity, marital status, reproductive history, and mental health treatment history.

#### Psychological flexibility

The Acceptance and Action Questionnaire (AAQ-II) [36] assesses psychological inflexibility and experiential avoidance—key constructs of the ACT framework. Within this 7-item measures, responses range from 1 (*never true*) to 7 (*always true*). Higher scores on the AAQ-II indicate less psychological flexibility. It evaluates a variety of concerns, such as being able to be grounded in the present moment, commitment of actions influenced by the participant’s values and being open to experiencing unwanted, negative feelings. Bond et al. [36] noted that cutoff scores for the AAQ-II ranging from 24 to 28 can indicate clinically significant distress. The AAQ-II has strong psychometric properties has more reliable outcomes across several samples than the AAQ-I.

#### Cognitive defusion

The Cognitive Fusion Questionnaire (CFQ) [37] assesses cognitive defusion, which is a core clinical process in ACT. Based on a 7-point scale, responses range from 1 (*never true*) to 7 (*always true*). Higher scores on the CFQ were related to increased fusion and prediction of distress. The CFQ has strong psychometric properties and showed stability across both clinical and non-clinical study samples.

#### Present-moment awareness and acceptance

The Philadelphia Mindfulness Scale (PHLMS) [38] assesses awareness and acceptance of thoughts and feelings in the present moment. On this 20-item measures, responses are on a 5-point Likert scale (1 = *never*, 2 = *rarely*, 3 = *sometimes*, 4 = *often*, and 5 = *very often*). Higher scores on the acceptance subscale of the PHLMS indicate higher levels of acceptance or willingness, while maintaining low levels of rumination. Higher scores on the awareness subscale of the PHLMS indicated higher levels of mindfulness. The PHLMS has good psychometric properties, indicating significant differences between clinical and nonclinical settings.

#### Perinatal distress and well-being

The Inventory of Depression and Anxiety Symptoms (IDAS) [39] assesses well-being and emotional distress (i.e., depression, anxiety). Within this 64-item measure, responses are on 5-point Likert scale assessing the extent to which the symptoms have been experienced within the last two weeks (1 = *not at all* to 5 = *extremely*). Higher scores on the IDAS indicated more distress and symptomatology. It calculates ten subscales including well-being, suicidality, lassitude, insomnia, appetite loss, appetite gain, ill temper, panic, social anxiety, and traumatic intrusions. It also has two total scores of general depression and dysphoria based on DSM-IV criteria for Major Depressive Disorder and Generalized Anxiety Disorder. The IDAS has strong psychometric properties given that it is correlated with BDI-II and BAI and effective in differentiating clinical and non-clinical participants [40]. It has been used in PMADS populations to screen women for perinatal distress [40]. In main analyses, we used the IDAS scales of well-being and dysphoria (referred to as “perinatal distress”) as our primary outcomes.

### Procedures

The institutional review board at Holy Family University approved this study. Electronic written consent was obtained. We used an online snowball sampling method on social media networking platform (i.e., Facebook) to recruit participants. We shared study information on Facebook groups for postpartum women, such as postpartum support groups, breastfeeding groups, and through direct messages. We shared information about the study using following text: “Postpartum moms face unique challenges and barriers when it comes to mental health, and we want to hear about the experiences of new moms so we can improve psychological treatment. If you are over 18 and have given birth in the past year, please take our survey {survey link here}.”

Potential participants who accessed the survey link first provided informed consent and then answered eligibility questions. Ineligible participants were not displayed any survey questions and provided with mental health resources. Eligible participants completed a de-identified electronic survey through Qualtrics at a single time point.

### Data analytic plan

We analyzed data using SPSS, version 26.0 (IBM, 2017). Prior to main analyses, we examined assumptions (normality, linearity, homoscedasticity, and independence)—all were met—and descriptive statistics. G*Power 3.1.7 [41, 42] indicated that the main analyses required 118 participants for power of .80 with a medium effect size (.15) and alpha set at .05. Therefore, given our sample size of ~220, primary analyses were sufficiently powered.

For Aim 1, we used SPSS 26.0 to conduct two separate hierarchical regression analyses for each outcome (i.e., well-being, emotional distress). In block 1, models included covariates of current mental health treatment (yes or no), income, number of children, and relationship status (partnered or single). For block 2, models included present-moment awareness, acceptance, and cognitive defusion to examine their unique contributions on outcomes. Block 3 included psychological flexibility to assess whether and to what extent it added unique variance to each outcome. Within the ACT framework, awareness, acceptance, and defusion are considered elements of psychological flexibility [43]. Given the theoretical overlap and correlations among variables, it was important to have psychological flexibility into a separate block.

For Aim 2, we used Hayes [44] PROCESS macro-Model 4 for SPSS to test whether psychological flexibility mediated the relationship between engagement in mental health treatment (yes/no) and each outcome (i.e., well-being, emotional distress). In each multiple mediation model, we entered engagement in mental health treatment as the predictor, psychological flexibility as the mediator, and well-being and emotional distress as the outcome. We present the findings for each step of mediation (path *a*, path *b*, path *c*, path *c’*, and path a*b). The indirect effect, from the predictor variable, through the hypothesized mediator, to the outcome variable, is the only requirement necessary to demonstrate mediation [45, 46]. We tested the indirect effect using bootstrap estimation approach with 1000 samples [47]. The 95% confidence intervals not containing zero indicate significant indirect effect [48]. We also reported the value of the total effect (path *c*) and attenuation of the direct effects after inclusion of the hypothesized mediators (path *c*’) for descriptive purposes.

## Results

### Participant characteristics

Table 1 presents participant characteristics including their socio-demographics, mental health history, and reproductive history (*N* = 266). Most participants were White (93.2%), married (94%), employed full-time (55.3%), and had earned a Bachelor’s degree or higher (87.6%). Mean annual combined household income was $132,205 (*SD* = $73,949; range = $0–500,000). Participants’ average age was 32.6 years (*SD* = 3.9; range = 21–44).

**Table 1:** Participant characteristics (*N* = 266).

Race	*n* (%)
White	256 (96.24%)
Black or African-American	3 (1.13%)
Bi/multiracial	5 (1.88%)
Asian or Pacific Islander	1 (0.38%)
Not Reported	1 (0.38%)
**Ethnicity**	
Non-Hispanic or Latino/Latina	259 (97.37%)
Hispanic or Latino/Latina	7 (2.63%)
**Marital Status**	
Single (not currently in a relationship)	5 (1.9%)
Unmarried and living with a partner	11 (4.1%)
Married	250 (94%)
**Age and Income**	***M* ± *SD* (Min–Max)**
Age	32.61 ± 4 (21–44)
Annual Household Income	$132,205.92 ± $73,949 ($0 - $500,000)
**Mental Health History**	***n* (%)**
Diagnosis of postpartum depression or anxiety (Y)	59 (22.2%)
History of treatment for emotional distress (Y)	95 (35.7%)
Currently in treatment for emotional distress (Y)	40 (15%)
**Reproductive Health History**	
History of fertility treatments (Y)	52 (19.5%)
History of pregnancy loss (Y)	71 (26.7%)
Any child admitted to neonatal intensive care (Y)	54 (20.3%)

*Note*. 100% of participants had given birth in the past year. 100% of these births resulted in a live birth (inclusion criteria).

### Preliminary analyses

Table 2 displays the descriptive statistics for study variables. Means of present-moment awareness, acceptance, cognitive defusion, psychological flexibility, well-being, and emotional distress fell into expected ranges per measures. In our sample, 135 participants (50.8%) endorsed elevated levels of emotional distress (i.e., dysphoria scores > 19.5), which exceeds estimated rates of emotional distress among postpartum women [49].

**Table 2:** Total scores for primary variables.

Variable, Measure, and Possible Range	*M ± SD*	*Sample Range*
**Outcomes**		
Well-Being (8–40)	23.35 ± 6.30	9–38
Emotional Distress (10–50)	22.30 ± 7.64	10–46
**Hypothesized Predictors**		
Present-Moment Awareness (10–50)	36.12 ± 5.90	11–50
Acceptance (10–50)	30.26 ± 7.76	12–50
Cognitive Defusion (7–49)	21.99 ± 8.92	7–49
Psychological Flexibility (7–49)	19.20 ± 7.21	7–43

*Note*. Higher well-being scores mean better well-being. Higher emotional distress scores indicate more depression and anxiety. Higher present-moment awareness scores mean greater mindfulness. Higher acceptance scores indicate more acceptance of internal experiences. Higher scores on cognitive defusion mean more fusion (negative) with thoughts. Higher flexibility scores mean more psychological inflexibility.

### Main analyses: Hierarchical regressions

#### Well-being (IDAS subscale)

A hierarchical regression indicated that the present-moment awareness, acceptance, cognitive defusion explained a significant portion of the variance in well-being, when controlling for number of children, current treatment engagement, income, and relationship status. Postpartum women who endorsed more present-moment awareness (*β* = .13; *p* = .023), acceptance (*β* = .17; *p* = .036), and cognitive defusion (*β* = -.46; *p* < .001) reported better well-being. When adding psychological flexibility into the model, awareness and acceptance were no longer significant. In this model, cognitive defusion (*β* = -.24; *p* = .031) and psychological flexibility (*β* = -.33; *p* = .003) explained a significant portion of variance in well-being (see Table 3).

**Table 3:** Factors explaining variance in well-being (*N* = 223).

**Model 1: Covariates**	**B**	**SE**	**β**	**t**	***p***
Relationship Status (Single/Partnered)	2.30	3.18	0.05	0.72	.470
Income	0.00	0.00	0.08	1.27	.205
Number of children	-0.43	0.50	-0.06	-0.86	.393
Currently in mental health treatment (Y/N)	4.21	1.14	0.24	3.70	< .001
**Model 2: Present-Moment Awareness, Acceptance, & Cognitive Defusion**					
Relationship Status (Single/Partnered)	1.16	2.64	0.02	0.44	.661
Income	0.00	0.00	-0.00	-0.06	.956
Number of children	-0.64	0.42	-0.01	-0.15	.879
Currently in mental health treatment (Y/N)	-0.27	1.03	-0.02	-0.26	.795
Present-Moment Awareness	0.14	0.06	0.13	2.29	.023
Acceptance	0.13	0.06	0.17	2.11	.036
Cognitive Defusion	-0.33	0.06	-0.46	-5.72	< .001
**Model 3: Psychological Flexibility**					
Relationship Status (Single/Partnered)	1.40	2.60	0.03	0.54	.591
Income	0.00	0.00	0.00	.03	.980
Number of children	-0.01	0.41	-0.00	-0.02	.981
Currently in mental health treatment (Y/N)	0.06	1.01	0.00	0.06	.954
Present-Moment Awareness	0.10	0.06	0.09	1.68	.094
Acceptance	0.08	0.07	0.10	1.21	.227
Cognitive Defusion	-0.17	0.08	-0.24	-2.17	.031
Psychology Flexibility	-0.28	0.10	-0.33	-2.97	.003

*Note*. Model 1: *F*(4, 219) = 4.22, *p* = .003, *R*^2^ = 7.2%; Model 2: *F*(7, 216) = 18.31, *p* < .001, *R*^2^ = 37.2% (*p* < .001); Model 3: *F*(8, 215) = 17.70, *p* < .001, *R*^2^ = 39.7%. Higher present-moment awareness scores mean greater mindfulness. Higher acceptance scores indicate more acceptance of internal experiences. Higher scores on cognitive defusion mean more fusion (negative) with thoughts. Higher flexibility scores mean more psychological inflexibility.

#### Perinatal distress (IDAS dysphoria scale)

A hierarchical regression indicated that acceptance and cognitive defusion explained a significant portion of the variance in emotional distress, when controlling for number of children, current treatment engagement, income, and relationship status. Postpartum women who endorsed more acceptance (*β* = -.32; *p* < .001) and cognitive defusion (*β* = .52; *p* < .001) endorsed less emotional distress. When adding psychological flexibility into the model, acceptance (*β* = -.28; *p* < .001), cognitive defusion (*β* = .40; *p* < .001), and psychological flexibility (*β* = .18; *p* = .047) explained significant variance in emotional distress (see Table 4).

**Table 4:** Factors explaining variance in emotional distress (*N* = 221).

**Model 1: Covariates**	**B**	**SE**	**β**	**t**	***p***
Relationship Status (Single/Partnered)	0.60	3.89	0.10	-.15	.878
Income	0.00	0.00	-0.11	-1.69	.093
Number of children	0.72	0.61	0.08	1.18	.241
Currently in mental health treatment (Y/N)	-5.09	1.41	-0.24	-3.61	< .001
**Model 2: Awareness, Acceptance, & Cognitive Defusion**					
Relationship Status (Single/Partnered)	2.08	2.62	0.04	0.79	.428
Income	0.00	0.00	0.02	0.33	.743
Number of children	0.23	0.42	0.03	0.56	.579
Currently in mental health treatment (Y/N)	-1.20	1.02	-0.06	-1.17	.224
Awareness	-0.04	0.06	-0.03	-0.63	.527
Acceptance	-0.32	0.06	-0.32	-5.03	< .001
Cognitive Defusion	0.46	0.06	0.52	7.93	< .001
**Model 3: Psychological Flexibility**					
Relationship Status (Single/Partnered)	1.91	2.60	0.03	0.73	.464
Income	0.00	0.00	0.01	0.29	.776
Number of children	0.20	0.41	0.02	0.49	.622
Currently in mental health treatment (Y/N)	-1.43	1.02	-0.07	-1.40	.163
Awareness	-0.01	0.06	-0.01	-0.19	.846
Acceptance	-0.29	0.07	-0.28	-4.24	< .001
Cognitive Defusion	0.35	0.08	0.40	4.50	< .001
Psychology Flexibility	0.19	0.10	0.18	1.99	.047

*Note*. Model 1: *F*(4, 217) = 4.26, *p* = .002, *R*^2^ = 7.3%; Model 2: *F*(7. 214) = 43.96, *p* < .001, *R*^2^ = 59.0%; Model 3: *F*(8, 213) = 39.50, *p* < .001, *R*^2^ = 59.7%. Higher present-moment awareness scores mean greater mindfulness. Higher acceptance scores indicate more acceptance of internal experiences. Higher scores on cognitive defusion mean more fusion (negative) with thoughts. Higher flexibility scores mean more psychological inflexibility.

### Main analysis: Mediation models

#### Well-being (IDAS subscale)

The *a* path indicated that current engagement in mental health treatment was associated with more psychological flexibility (*b* = -7.58, SE = 1.20, *p* < .001, CI = [-9.95, -5.22]). For the *b* path, increases in psychological flexibility was associated with better well-being (*b* = -5.15, SE = 0.05, *p* < .001 CI = [-0.61, -0.42]). Path *c* was significant, (*b* = 4.15, SE = 1.10, *p* < .001, CI = [1.99, 6.32]) indicating that engagement in mental health treatment was positively associated with well-being (total effect).

**Fig 1:**
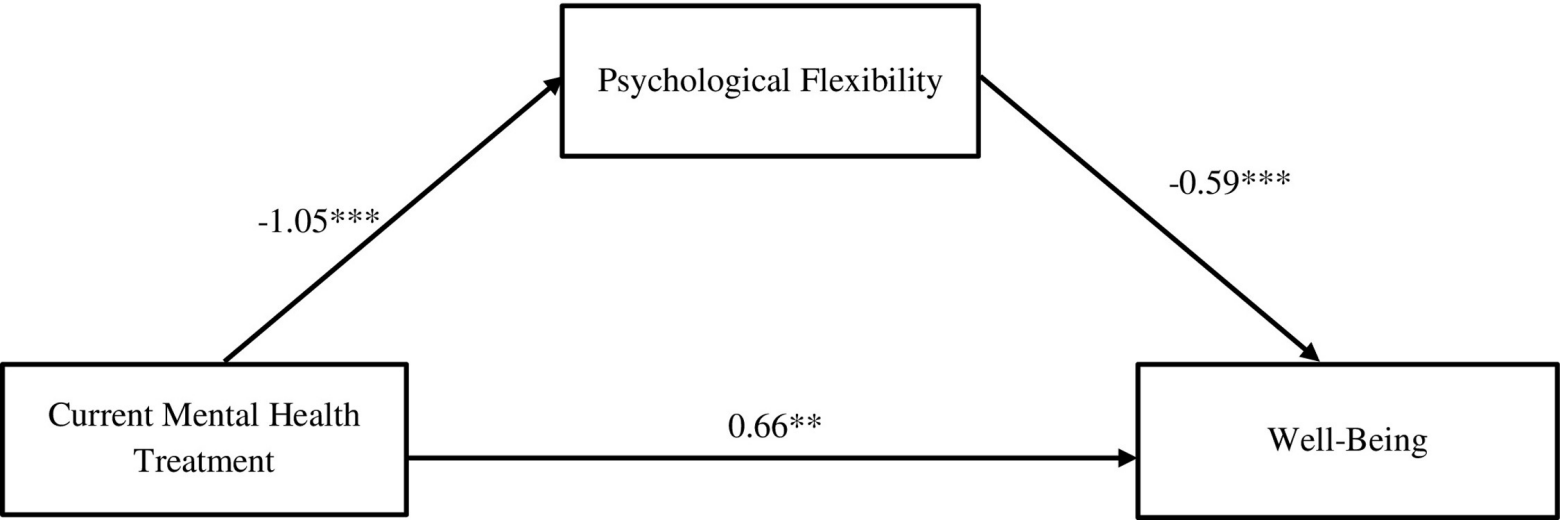
Mediation model demonstrating the effect of psychological flexibility on well-being. Paths specify standardized coefficients, *p < .05; ** *p* < .01; ***p < .001, path *c*’: *b* = 0.25, *p* = .803. Solid lines represent significant pathways.

When psychological flexibility was included in the model, the relationship between mental health treatment and well-being (path *c’*) was no longer significant, (*b* = 0.25, SE = 0.98, *p* = .803), which indicates full mediation. The indirect effect (path a*b) of mental health treatment on well-being through psychological flexibility was significant (*b* = 3.91, SE = 0.66, CI = [2.69, 5.30]) (see Fig 1). In other words, the relationship between mental health treatment and well-being was explained by psychological flexibility.

#### Emotional distress (IDAS dysphoria scale)

The *a* path indicated that current engagement in mental health treatment was associated with less emotional distress (*b* = -7.59, SE = 1.21, *p* < .001, CI = [-9.98, -5.20]). For the *b* path, more psychological flexibility was associated with better well-being (*b* = 0.76, SE = 0.05, *p* < .001 CI = [0.65, 0.86]). Path *c* was significant, (*b* = -4.87, SE = 1.36, *p* < .001, CI = [-7.54, -2.20]) indicating that engagement in mental health treatment was associated with less emotional distress (total effect).

**Fig 2:**
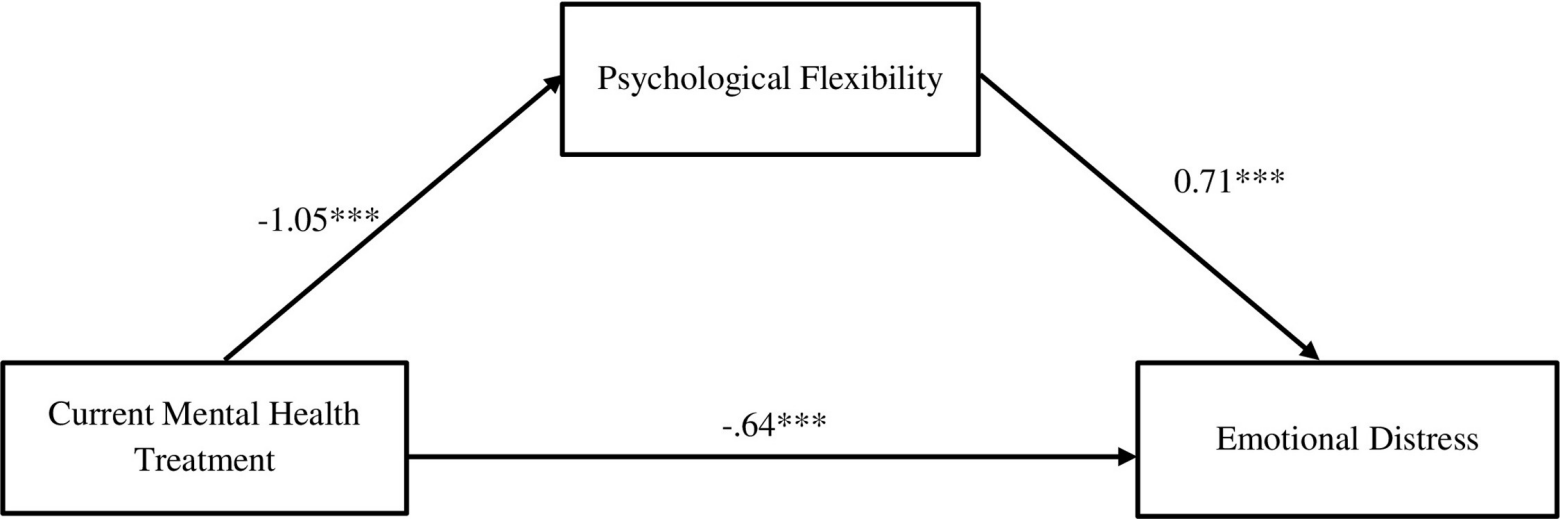
Mediation model demonstrating the effect of psychological flexibility on emotional distress. Paths specify standardized coefficients, *p < .05; ** *p* < .01; ***p < .001, path *c*’: *b* = 0.87, *p* = .421. Solid lines represent significant pathways.

When psychological flexibility was included in the model, the relationship between mental health treatment and well-being (path *c’*) was no longer significant, (*b* = 0.87, SE = 1.08, *p* = .420), indicating full mediation. The indirect effect (path a*b) of mental health treatment on well-being through psychological flexibility was significant (*b* = -5.74, SE = 1.08, CI = [-1.26, 3.00]) (see Fig 2). In other words, the relationship between mental health treatment and emotional distress was explained by psychological flexibility.

## Discussion

Although 10–20% of women experience PMADs [5], current psychosocial interventions are limited. Recent literature has indicated that third-wave cognitive-behavioral approaches may be a promising way to address this unmet clinical need. Prior to developing tailored acceptance-based interventions, an important first step is to examine whether the underlying treatment processes are related to postpartum outcomes. We investigated whether acceptance-based process variables (e.g., present-moment awareness, acceptance, cognitive defusion, and psychological flexibility) explained variance in well-being and emotional distress among postpartum mothers. We also examined whether psychological flexibility mediated the relationship between treatment engagement and postpartum outcomes (i.e., well-being, emotional distress). Below we describe our findings and their research and clinical implications.

Findings revealed that women who reported more awareness, acceptance, and cognitive defusion endorsed better well-being, even when controlling for known covariates (i.e., relationship status, income, number of children, engagement in mental health treatment). In addition, women who endorsed more acceptance and cognitive defusion (but not awareness) reported less emotional distress, when controlling for the same covariates. Together, findings suggest that mindfulness (awareness and/or acceptance) and cognitive defusion are important treatment targets to enhance postpartum outcomes. Notably, when we added psychological flexibility into each model, it explained a unique portion of the variance in well-being and emotional distress. Only one other study, to date, has examined the role of psychological flexibility among postpartum women [24]. Monteiro and colleagues [24] indicated that women without symptoms of depression and anxiety endorsed higher levels of psychological flexibility. Our findings extend this work given that our work indicates that: (1) awareness, acceptance, and cognitive defusion, in addition to psychological flexibility, are relevant to both well-being and emotional distress; (2) these acceptance-based process variables contribute to variability in these outcomes; and (3) variables are relevant across all postpartum women, not just those at risk for depression and anxiety.

Our findings also indicated that psychological flexibility mediated the relationship between mental health treatment and outcomes (i.e., wellbeing and emotional distress). Prior work investigating other populations of women—i.e., survivors of interpersonal violence, breast cancer survivors, women in the community experiencing stress—indicated that psychological flexibility protected against or helped reduce depression, anxiety, and post-traumatic stress disorder [50–52]. In addition, ACT intervention studies indicate that psychological flexibility is the core mechanism that helps to improve anxiety, depression, psychosis, and chronic pain [53, 54]. Consistent with this literature, our findings suggest that psychological flexibility may be a transdiagnostic mechanism that can help improve well-being depression, and anxiety in postpartum women. Notably, women in our sample participated in a variety of mental health treatments (individual, group, medication) suggesting psychological flexibility could be targeted across therapeutic modalities and therefore applicable across psychiatry, psychology, and social work. Focusing on treatment mechanisms rather than different therapeutic approaches could be more efficient, cost-effective, and effective in improving postpartum outcomes.

### Strengths, limitations, and future directions

The present study has many strengths. First, we had a large sample, which ensured that analyses were powered. Second, we focused on understudied acceptance-based processes in this population, which is a first step to informing new perinatal-specific interventions. Third, we used well-validated measures within a general population, which helps to increase variability in responses. There are also several important limitations to note. First, our sample was predominantly White (96.24%) and endorsed higher than average incomes (mean annual household income = 132,205.92±$73,949), which limits generalizability of findings across demographic groups and cultural contexts. The fact that we recruited through social media networks and that the survey was online may have introduced selection bias, as we primarily targeted individuals who were active on social media and engaged in online support groups, suggesting that our sample may not represent the broader population of postpartum women. Future research should examine these process variables in larger, more diverse samples, ideally recruited from a range of settings rather than solely using online snowball sampling recruitment. A larger, more diverse sample would allow for testing of interactions as well as subgroup analyses and enhance generalizability. Second, this was a cross-sectional study design; therefore, causality cannot be assumed and findings should be interpreted with caution. This point is important for the mediation models given that they tend to assume temporal precedence and that confounds are controlled for in the models. Although theoretically and conceptually, psychological flexibility is a hypothesized mechanism of change in interventions (such as ACT), the variables may be reciprocally related in our study and/or may be related to a confounding variable. Future work is needed to test mediation analyses longitudinally and in context of a clinical trial to determine whether psychological flexibility serves as a mechanism of change when being directly targeted. Third, there are limitations with the use of self-report measures, such as social desirability bias and response bias. Moreover, because women self-reported treatment engagement, we do not have information on the interventions and skills they were taught in their mental health treatments. Future work may include clinician-administered assessments in addition to self-report measures to increase the validity of findings. Fourth, we focused on depression and anxiety as the primary outcomes. More work is needed to examine how psychological flexibility impacts important postpartum outcomes such as sleep, maternal-child attachment, and partner/family relationships. Fifth, we only examined a few components of psychological flexibility. Additional research is needed to determine how values and commitment to action can help promote psychological flexibility and, in turn, behavior change. Finally, our study focused on self-report quantitative data. Qualitative and mixed methods data would help inform the development of acceptance-based interventions for perinatal women. These methodological approaches are key for understanding gaps in current treatment and how to make treatment for accessible and tailored for this population.

### Clinical implications

Our findings have important clinical implications. They suggest that improving awareness, acceptance, cognitive defusion, and psychological flexibility within postpartum women can enhance well-being and reduce distress. Clinicians can use mindfulness and defusion strategies to help mothers identify and create distance from self-critical thoughts (e.g., “I’m a bad mom”) and feelings of guilt. Recognizing and changing one’s relationship to these internal experiences may help promote maternal engagement in valued behaviors (e.g., time with baby, self-care) [27–29]. These interventions can be delivered in a brief integrated care format or through referrals to longer term individual outpatient care. In fact, group ACT interventions for postpartum women have been developed in clinical settings to help normalize postpartum experiences and address the feelings of social isolation that can come with the transition to motherhood [27–29]. Group interventions may increase access and engagement and may be more cost-effective. However, no fully powered randomized clinical trials testing the efficacy-effectiveness of ACT in either group or individual formats within perinatal populations have been conducted. However, recently developed ACT interventions for perinatal women demonstrated acceptability, feasibility, and preliminary effectiveness in reducing distress [30–34]. Additional mixed methods research (e.g., qualitative interviews with women with PMADs; focus groups with providers; longitudinal assessment of acceptance-based mechanisms and psychosocial outcomes) is needed to inform the development of new interventions (pilot, feasibility, efficacy trials). It would help to identify in-depth challenges of this population, barriers and facilitators to engagement, and suggestions for enhancing intervention feasibility and efficacy-effectiveness.

### Conclusions

The transition to motherhood is filled with complex emotions, unexpected changes, and new roles and responsibilities. With up to 20% of women experience depression and/or anxiety symptoms during the postpartum period, improved interventions are needed. Our findings indicated that awareness, acceptance, cognitive defusion, and psychological flexibility contributed to well-being and/or emotional distress among postpartum women. Further, psychological flexibility served as a mediator between current mental health treatment and postpartum outcomes. Licensed mental health providers who work with perinatal women may consider incorporating and tailor ACT skills (e.g., defusion, mindfulness) to help enhance psychological flexibility and, in turn, reduce distress. More research is necessary to develop, test, and implement acceptance-based interventions for diverse perinatal women.
